# Prevalence and clinical significance of early endotoxin activity in septic shock patients

**DOI:** 10.1186/cc14128

**Published:** 2015-03-16

**Authors:** M Bottiroli, R Pinciroli, G Monti, M Mininni, G Casella, R Fumagalli

**Affiliations:** 1 Anestesia Rianimazione 3, A.O. Niguarda, Milan, Italy; 2 Anestesia Rianimazione 1, A.O. Niguarda, Milan, Italy

## Introduction

The endotoxin activity (EA) assay is a useful test to risk stratify critically ill patients and assess for Gram-negative (GN) infection. However, the prevalence and significance of early high levels of EA in patients with septic shock (SS) has yet to be elucidated.

## Methods

We designed a prospective observational study including adult patients with clinically diagnosed SS. EA was measured on arterial blood by a chemiluminescent assay within the first 24 hours from SS diagnosis. The finding of an EA value ≥0.6 was used as the cutoff for test positivity, as described elsewhere. In addition, laboratory, microbiological and clinical data were collected at inclusion. In-hospital follow-up was also conducted.

## Results

**Figure 1:**
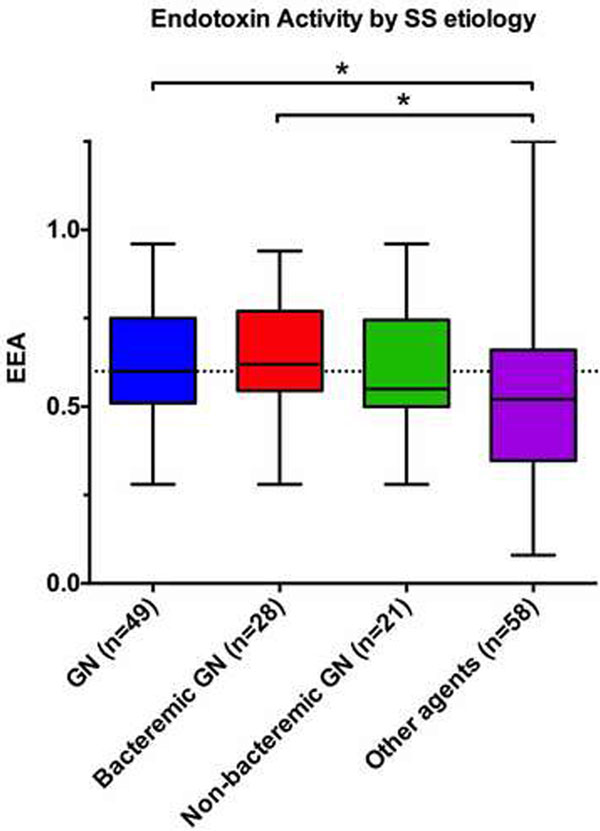


A total of 107 consecutive patients were included. The overall median EA was 0.56 (0.44 to 0.71), with 46/107 (43%) patients testing positive for elevated EA (≥0.6). GN species were identified in microbial cultures as the infective etiology in 49/107 (46%) patients, of which 28 (57%) developed bacteremia. GN infections were associated with higher levels of EA compared with other microbial causatives (0.61 (0.52 to 0.77) vs. 0.52 (0.38 to 0.64), *P *= 0.021). Patients with EA ≥0.6 showed significantly higher lactate levels (2 (1 to 3) vs. 3.8 (1.7 to 6.4), *P *= 0.01), Sequential Organ Failure Assessment (9 (6 to 12) vs. 10 (8 to 14), *P *= 0.04) and inotropic score (20 (5 to 50) vs. 50 (16 to 100), *P *= 0.003) at inclusion. See Figure 1.

## Conclusion

Elevated EA is a common finding in SS patients. In patients developing SS from a GN infection, higher levels of endotoxin activity could be measured within 24 hours. Furthermore, in our study, EA ≥0.6 identified a subgroup of subjects at greater risk for worse clinical outcomes. We therefore propose use of the EA assay for the early identification and risk stratification of SS patients.

